# Evolutionary conservation of putative suicidality-related risk genes that produce diminished motivation corrected by clozapine, lithium and antidepressants

**DOI:** 10.3389/fpsyt.2024.1341735

**Published:** 2024-02-01

**Authors:** Titilade Ajayi, Alicia Thomas, Marko Nikolic, Lauryn Henderson, Alexa Zaheri, Donard S. Dwyer

**Affiliations:** ^1^ Department of Pharmacology, Toxicology and Neuroscience, LSU Health Shreveport, Shreveport, LA, United States; ^2^ Department of Psychiatry and Behavioral Medicine, LSU Health Shreveport, Shreveport, LA, United States

**Keywords:** antidepressants, *C. elegans*, clozapine, diminished motivation, lithium, suicide

## Abstract

**Background:**

Genome wide association studies (GWAS) and candidate gene analyses have identified genetic variants and genes that may increase the risk for suicidal thoughts and behaviors (STBs). Important unresolved issues surround these tentative risk variants such as the characteristics of the associated genes and how they might elicit STBs.

**Methods:**

Putative suicidality-related risk genes (PSRGs) were identified by comprehensive literature search and were characterized with respect to evolutionary conservation, participation in gene interaction networks and associated phenotypes. Evolutionary conservation was established with database searches and BLASTP queries, whereas gene-gene interactions were ascertained with GeneMANIA. We then examined whether mutations in risk-gene counterparts in *C. elegans* produced a diminished motivation phenotype previously connected to suicide risk factors.

**Results and conclusions:**

From the analysis, 105 risk-gene candidates were identified and found to be: 1) highly conserved during evolution, 2) enriched for essential genes, 3) involved in significant gene-gene interactions, and 4) associated with psychiatric disorders, metabolic disturbances and asthma/allergy. Evaluation of 17 mutant strains with loss-of-function/deletion mutations in PSRG orthologs revealed that 11 mutants showed significant evidence of diminished motivation that manifested as immobility in a foraging assay. Immobility was corrected in some or all of the mutants with clozapine, lithium and tricyclic antidepressant drugs. In addition, 5-HT2 receptor and muscarinic receptor antagonists restored goal-directed behavior in most or all of the mutants. These studies increase confidence in the validity of the PSRGs and provide initial clues about possible mechanisms that mediate STBs.

## Introduction

According to statistics from the World Health Organization, nearly 800,000 people die by suicide each year worldwide (1). For every person who takes their own life, there are roughly 200 others who seriously considered killing themselves in a given year (2). This amounts to roughly 10 million people in the US annually who have experienced suicidal thoughts and behaviors (STBs) (2).

STBs reflect highly complex and dynamic interactions between psychiatric conditions, such as major depression, internal factors, e.g., feelings of hopelessness, genetic vulnerability, adverse childhood experiences (ACEs) and external stressors (3–7). Genetic contributions to STBs have been implicated by studying heritability in families and twins (8–11), and by establishing the presence of common risk factors in genome-wide analyses (12, 13). The heritability of suicide is estimated in the range of 35-50% (9, 10). Consequently, genome-wide association studies (GWAS) and candidate gene analyses have sought to identify genetic variants that increase risk for suicide, and a number of promising candidates have emerged (13–32). It is not known whether suicide risk genes are a unique subset of psychiatric risk genes and further, whether some identified thus far represent false positives. Additional studies are needed to address these issues. Toward this end, we have compiled a list of suicide risk variants from GWAS and candidate gene analyses. The goal was to characterize the associated genes with respect to: 1) their degree of evolutionary conservation, 2) related phenotypes in *Caenorhabditis elegans* and humans, 3) their participation in gene interaction networks and 4) functional activity in relevant *C. elegans* behavioral models. Based on previous studies of genetic risk variants for schizophrenia, bipolar disorder and major depression (33–35), we hypothesized that the putative suicide risk genes would be highly conserved during evolution and enriched for genes essential for life. Moreover, suicide risk genes may be more highly connected in gene-gene interaction networks than similar-sized lists of randomly-selected genes. This is important because a high level of network connectivity among putative risk genes may confirm their relevance to a psychiatric disorder (36).

Mutation of a single gene is unlikely by itself to trigger the full spectrum of STBs. Instead, risk variants may affect relevant endophenotypes such as impulsivity and aggression, HPA axis dysfunction and serotonergic activity (37, 38). Furthermore, some endophenotypes may be evolutionarily conserved and constitute protophenotypes, as defined previously (39), that can be studied experimentally in animal models.

We reasoned that mutations in some risk genes related to suicidality might produce relevant protophenotypes in *C. elegans* with a focus on diminished motivation states. Diminished motivation to search for food is detected as immobility in a foraging assay (40) and is conceptually similar to the immobility measured in the forced swim test – an established rodent model of depressive behavior (41). The failure of certain *C elegans* mutants to engage in goal-directed behavior following food deprivation has previously been likened to suicidal behavior because animals remain immobile for more than 10 days or until they die without trying to locate food or escape from harmful conditions (39). Reduced goal-directed behavior reflects diminished motivation and resembles anhedonia and hopelessness (42) – two major risk factors for depression and STBs. In *C. elegans*, motivation to forage for food is regulated by insulin signaling (40), which is significant because defective insulin signaling in humans is not only associated with diabetes mellitus, but also apathy (43), depression (44) and suicide (45). Finally, we hypothesized that drugs shown to reduce suicidal behavior in humans, including clozapine (46), lithium (47, 48), and antidepressants (49), would likewise improve diminished motivation in animals with mutations in suicide risk-gene counterparts.

Here, we report the extensive characterization of possible suicide risk-gene candidates in terms of their evolutionary conservation, participation in gene-gene interactions and role in goal-directed (motivated) behavior.

## Materials and methods

### Compilation and characterization of reported risk genes for suicidality

We identified a large representative sample of possible suicide risk genes (105) implicated in GWAS and candidate gene analyses identified in PubMed^®^ searches (13–32). Thirteen of the 20 studies were derived from GWAS published between 2010-2020. Seven studies, providing a total of 10 genes, were candidate gene analyses, which ensured that the focus was not limited exclusively to GWAS results. Overall, the studies included subjects who attempted suicide or had suicidal ideation/behavior across diagnostic categories (depression, bipolar disorder, and schizophrenia). Combining risk genes across diagnoses and STBs can raise issues. For example, genetic factors do not faithfully align in relation to suicidal ideation, suicide attempts, and suicide – they are not all the same (50, 51). Furthermore, mixing suicide-related phenotypes may reduce the ability to resolve genetic influences (52). Despite this caveat, we wished to gain the broadest possible perspective on genetic risk associated with all aspects of STBs. By examining a larger pool of PSRGs, we may be able to detect patterns of gene function and phenotypes that provide initial clues missed by a more circumspect approach. Therefore, the set of genes listed in **Supplementary Table 1** should be considered putative suicidality-related risk genes (PSRGs). In view of the preliminary nature of the PSRGs, we sought to further verify their status by evaluating network connectivity and production of relevant phenotypes.

We searched for functional counterparts in *C. elegans* as described previously (35). Briefly, we started by checking for consensus orthologs listed in the Ensembl database (maintained by the European Molecular Biology Laboratory’s European Bioinformatics Institute). In cases where no ortholog was listed, we performed BLASTP searches of WormBase with the longest amino acid sequence of the human risk gene and with the E-value threshold set to +2. Top hits were chosen based on established criteria (35) and follow-up searches with sequences of orthologs from other species served to confirm the identity of the functional counterparts in *C. elegans*. The orthologs/functional counterparts of the human PSRGs are listed in **Supplementary Table 1**. Because there is extensive phenotype data for most *C. elegans* genes, this information was included in the Table. In addition, we searched the Ensembl database for human phenotypes, which are also listed in **Supplementary Table 1** (Human Phenotypes Ensembl.org).

Phenotype analysis was performed with keyword searches (e.g., lethal or aldicarb) of the risk-gene spreadsheet and unique entries were quantified. We considered genes to be essential if mutations caused lethality or sterility after the convention of Kemphues (53). Human phenotypes associated with genetic variation were searched in a similar manner after identification of recurring themes that were used as search terms.

### Characterization of gene-gene interactions

To characterize genetic interactions among the PSRGs in comparison to a similar-sized sample of randomly selected genes, we used Molbiotools and GeneMANIA (54) as described previously (35). The settings used for the analysis were restricted to “Genetic Interactions” with “Max resultant genes” (additional genes) and “Max resultant attributes” set to 0. The list of PSRGs was submitted to GeneMANIA and the number of links per gene was obtained from this analysis. This measure was used to compare random vs. risk genes. Confidence intervals for the random gene data were derived by analyzing four separate lists of 105 randomly-selected genes, obtaining the number of links per gene for each list and then determining the mean and standard deviation for the control (random) data.

### *C. elegans* strains

We hypothesized that mutations in the *C. elegans* counterparts of PSRGs may produce relevant phenotypes, namely diminished motivation to search for food (39, 42). As a representative sample, we selected 17 strains for behavioral testing based on these criteria: mutations were not lethal, they did not cause severe phenotypes that interfered with testing and mutant strains were available from the Caenorhabditis Genetics Center (CGC). **Table 1** shows details about the panel of strains, which were either null, loss-of-function or deletion mutants [CGC database and ref. (56)].

**Table 1:** Human PSRGs and Relevant Mutant Stains of C. elegans*.

Human PSRG	Description	*C. elegans* counterpart (allele)	Strain designation
CPLX1	complexin	*cpx-1(ok1552)*	RB1367
DCC	DCC netrin 1 receptor	*unc-40(e271)*	CB271
GALNT10	N-acetylgalactosaminyl- transferase 10	*gly-10(ok2439)*	RB1888
GRIA1	glutamate ionotropic receptor AMPA type subunit 1	*glr-1(n2461)*	KP4
GRM5	glutamate metabotropic receptor 5	*mgl-2(tm355):* *mgl-1(tm1811)*	DA2250
HIPK2/3	homeodomain interacting protein kinase 2/3	*hpk-1(pk1393)*	EK273
IGSF9B	immunoglobulin superfamily member 9B	*igcm-2(ok1527)*	RB1360
LHX6	LIM homeobox 6	*lim-4(yz12)*	JY359
LHX6	LIM homeobox 6	*ttx-3(ot22)*	OH161
PDE4B	phosphodiesterase 4B	*pde-4(ok1290)*	RB1231
PDPK1	3-phosphoinositide-dependent protein kinase 1	*pdk-1(sa680)*	JT9609
RBFOX1	RNA binding fox-1 homolog 1	*fox-1(e2643)*	CB5380
RGS6	regulator of G protein signaling 6	*egl-10(md176)*	MT8504
STK33/BRSK2	serine/threonine kinase 33 BR serine/threonine kinase 2	*sad-1(ky289)*	CX5156
TBX20	T-box transcription factor 20	*tbx-2(ut180)*	JC1970
CNR1	cannabinoid receptor 1	*npr-19(ok2068)*	RB1668
SLC35D1	UDP-N-acetylgalactosamine transporter	*sqv-7(n2839)*	MT7562

*Human genes are listed here along with a brief description of their function. The *C. elegans* homologous equivalents are shown with the mutant allele in parentheses. The strain expressing that allele is listed in the last column. The bottom two strains were tested because they (MT7562) either related to a main member of the list, e.g., the UDP-N-acetylgalactosamine transporter SQV-7 [SLC35D1] furnishes the substrate for GALNT10/GLY-10 or they (RB1668) bore mutations in a cannabinoid receptor, NPR-19 [CNR1], independently linked to suicidal ideation (55), although not a risk gene per se.

### Immobility assay and drug studies

We sought to determine if functional loss of the PSRG counterparts in *C. elegans* caused diminished motivation to forage as described previously (40). Additional details are included in the **Supplementary Materials**. Young adult animals (~ 20-25 per plate) from different mutant strains were first exposed to one of three conditions – control buffer (dilute acetic acid), dimethyl sulfoxide (DMSO) in buffer (1% final concentration on plates) or DMSO/buffer plus drug – on plates with food for 1.5 hr. Next, they were transferred to large plates (100 mm) that lacked bacteria, but that matched the initial conditions for DMSO, drug, etc. We then evaluated movement with a microscope after 2.5-3 hr of food deprivation. Animals were counted as moving if they traversed more than half their body length in either direction during a 5-sec observation period. We counted the number of animals moving and the total number on the plate to calculate the % Moving.

For the drug rescue studies, we used relevant psychotropic drugs. Stock solutions were prepared in DMSO at a concentration of 40 mM, except for lithium, which was prepared in water at 330 mM and atropine, also prepared in water at 99 mM. Drugs were diluted in 10^-4^ M acetic acid to the desired final concentration (generally 160 μM for antipsychotics/antidepressants, 6.7 mM for lithium and 2 mM for atropine). The choices for drug concentrations are justified in the **Supplementary Materials**. Control and drug plates both contained DMSO at the same final 1% concentration. The plates were allowed to dry and equilibrate for 2-4 hr at room temperature prior to use in the assay.

### Statistical analyses

Statistical analysis of the evolutionary conservation of risk genes and the degree of gene-gene interactions obtained from GeneMANIA has been described before (33, 35). We used whole genome comparisons for the conservation data across species and partial genome comparisons for the phenotype analysis of genes considered Essential, Lethal or to affect Lifespan. Because the raw data for these genome comparisons are non-parametric, we used chi-square analysis (33).

To determine background levels of genetic interactions, we generated four lists each with 105 randomly-selected human genes using Molbiotools as described before (33, 35). The random gene lists were evaluated for gene interactions with other members of that random list using GeneMANIA and the number of links (interactions) per gene was calculated for each gene included in the interaction database. PSRGs were evaluated for gene-gene interactions in the same way. The number of links per gene from the four random lists was averaged and the standard deviation was obtained to derive a confidence interval equal to 4 times the standard deviation (p < 0.0001).

For the immobility assay, each experiment (control [DMSO] vs. drug concentration) was repeated at least three times; in most cases N ≥ 4. Each strain was tested in separate experiments and drugs were likewise evaluated individually with each strain. When sufficient data from all repeat experiments with a particular drug/condition and mutant strain were collected, they were averaged and the standard deviation was calculated. Student’s t-test was used to determine whether differences between each condition (e.g., drug vs. DMSO control) – obtained from individual experiments – were significant at p < 0.05.

## Results

### Putative suicidality-related risk genes are evolutionarily conserved

**Figure 1:**
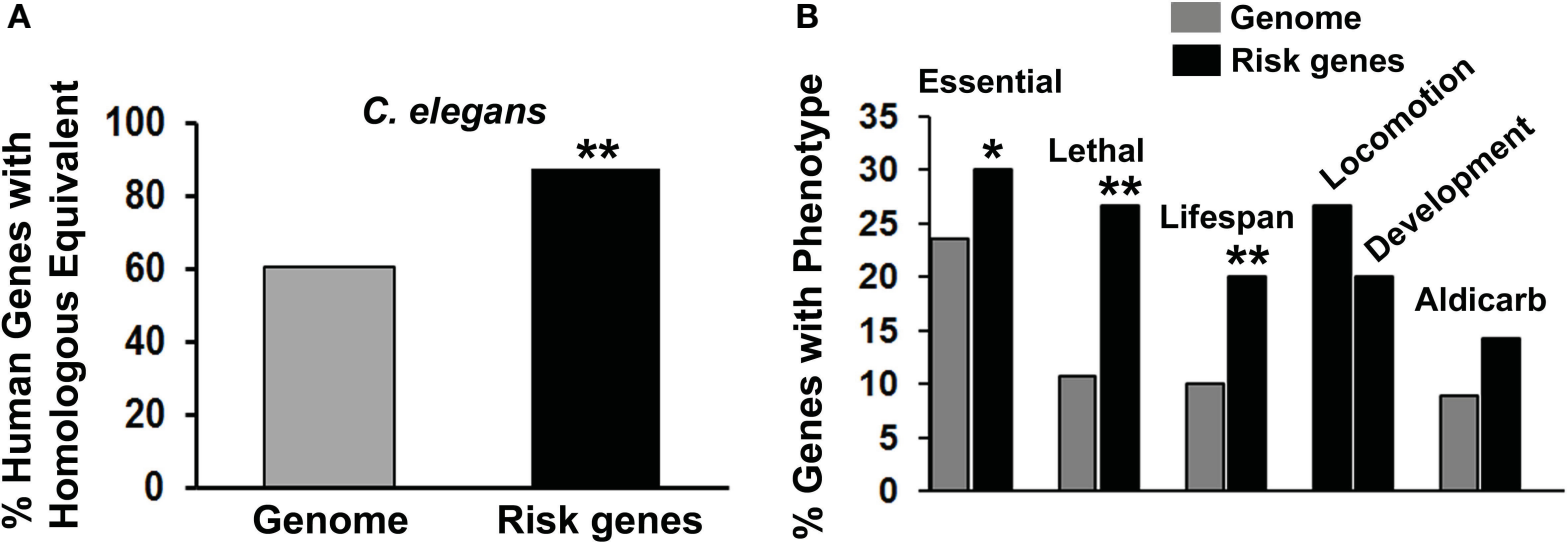
Characterization of suicide risk genes. **(A)** We compared the frequency of finding counterparts of human genomic genes in the *C. elegans* genome (gray bar) with the frequency of finding counterparts of the PSRGs in *C. elegans* (black bar). When analyzed separately, 87.3% of genes derived from GWAS were conserved in *C. elegans*, whereas that number was essentially the same – 90% – from candidate gene analysis. Significant differences (**p < 0.01) between these comparisons have been indicated by asterisks. **(B)** The phenotypes associated with PSRG counterparts in *C. elegans* were tabulated (black bars) and compared with the frequency of phenotypes observed in genome-wide analyses of gene function (gray bars). For the chi-square tests, the Genome control data from whole or partial genome analyses were: 4645/19727 (23.5%) essential genes, 264/2445 (10.8%) lethal genes, 1876/18496 (10.1%) genes that affect lifespan and 185/2072 (8.9%) genes with aldicarb sensitivity. Comparable genomic analysis is not available for Locomotion or Development phenotypes, so only the raw data are shown. Phenotypes are labelled and asterisks indicate p values < 0.05* and < 0.01**.

From 20 published studies, we compiled a list of 105 PSRGs implicated in various suicidal behaviors (**Supplementary Table 1**). Although we have inevitably missed some possible risk genes in the literature, this compilation is the largest yet published to our knowledge. Several genes in these studies were duplicated: ABI3BP, CTNNA3 and HIPK2/3. As hypothesized, PSRGs were significantly conserved during evolution with 87.6% of the human risk genes having a counterpart in *C. elegans* versus only 60.8% of the genes in the entire human genome (57) (**Figure 1A**). This degree of conservation is similar to that observed for risk genes associated with depression, bipolar disorder and schizophrenia.

### Enrichment for essential genes and other phenotypes

Evaluation of phenotypes associated with the risk gene counterparts revealed that the PSRGs are significantly enriched for essential genes, lethality and genes that influence lifespan (**Figure 1B**). Genes that affected locomotion and development appeared to be over-represented, whereas the aldicarb (cholinesterase inhibitor) phenotype (15 genes) showed a trend toward enrichment. Other notable *C. elegans* phenotypes included: dauer formation (13 genes), which is regulated by insulin signaling, imipramine (5 genes), serotonin (4 genes) and egg laying (15 genes), which is controlled by acetylcholine and serotonin.

Similar analysis of phenotypes observed in humans as the result of variation in the PSRGs was also informative. Three major clusters were identified (see **Table 2**). The first unsurprisingly included the psychiatric disorders depression, schizophrenia and bipolar disorder. The second cluster comprised metabolic phenotypes such as diabetes mellitus, weight and cholesterol. The third included bronchial asthma and allergic disorders. Sleep and autism were also recurring themes and were linked to 19 and 15 of the PSRGs, respectively. Some of the genes were very pleiotropic; for example, CTNNA3, RERE, SMAD3 and RBFOX1 contributed to all three major phenotype clusters, while DCC, GALNT10, NKAIN2, PDE4B and RGS6 were associated with 6 or 7 different phenotypes.

**Table 2:** Phenotypes and Conditions Associated with PSRGs*.

Phenotype Clusters	Phenotype/ Condition	Suicide Risk Genes
**Psychiatric Disorder** **Cluster**	Depression (24)	**RGS6**; **GRIN2B**; (**PDE4B-INSL5**); (ARL5B-**CACNB2**); **RERE**; **NKAIN2**; **ZCCHC2; RBFOX1** DCC; UBE2H; (PREX1-ARFGEF2); DPP10; CACNA1C; ALX4; ITIH3; ITIH4; LUZP2; SPAG17; PCSK5; TMEM132C; RGS10; STK3; GRM5
	Schizophrenia (23)	(**GRIA1**-**GALNT10**-LARP1); **RGS6**; (**PDE4B-INSL5**); (ARL5B-**CACNB2**); **RERE**; **NKAIN2**; **CTNNA3; RBFOX1** IGSF9B; DCC; LRRIQ3; KCNMB2; LUZP2; DLG2; PCSK5; CACNA1C; CKB; LRRTM4; CADM2; TSPAN18; ITIH3; ITIH4
	Bipolar disorder (23)	**RGS6**; (**PDE4B-INSL5**); **CACNB2**; **SMAD3**; **RERE**; **NKAIN2**; **ZCCHC2; RBFOX1** IGSF9B; DCC; PRSS3; ARNT2; LRRIQ3; KCNMB2; GXYLT1; NTRK2; CACNA2D4; PCSK5; CACNA1C; TMEM132C; CADM2; ITIH3; ITIH4
**Metabolic** **Cluster**	Weight (21)	(**GRIA1**-**GALNT10**-LARP1); **RGS6**; (**PDE4B-INSL5**); **SMAD3**; **NKAIN2**; **CTNNA3** TBX20; DCC; ABI3BP; ARFGEF2; CAPN13; LUZP2; BACE1; NHS; NTRK2; DLG2; PCSK5; TMEM132C; CADM2; GNAS
	Cholesterol (25)	(**GRIA1**-**GALNT10**-LARP1); (**PDE4B-INSL5**); **CACNB2**; **SMAD3**; **NKAIN2**; **ANXA2**; **CTNNA3; RBFOX1** FOXB1; DPP10; SLCA4; KNMB2; LUZP2; NTRK2; (FAH-ARNT2); GNAS; CACNA1D; CACNA2D4; DLG2; PCSK5; FAM110C; SH3YL1; TMEM132C; GSK3B
	Waist circumference (17)	**RGS6**; **SMAD3**; **NKAIN2**; **ZCCHC2**; **CTNNA3** TBX20; GNAS; (PREX1-ARFGEF2); SPAG17; NTRK2; DLG2; NAV2; SH3YL1; TMEM231C; CADM2; ITIH3; ITIH4
	Diabetes (17)	(**GRIA1**-**GALNT10**-LARP1); (ARL5B-**CACNB2**); **ANXA2; RBFOX1** DCC; HIPK2; (FAH-ARNT2); GNAS; STK32B; ABI3BP; CAPN13; NTRK2; PRDM16; DLG2; TMEM132C; IMPA2
**Inflammatory** **Cluster**	Asthma (15)	**GRIN2B**; **SMAD3**; **RERE**; **ZCCHC2**; **ANXA2**; **CTNNA3; RBFOX1** FOXB1; IAPP; ABI3BP; LRRIQ3; TBL1XR1; ING5; NAV2; TRDN
	Allergy (6)	**GRIN2B**; **SMAD3**; **RERE; RBFOX1** LRRIQ3; PRDM16
**Other** **Neuro-psychiatric** **Cluster**	Sleep (20)	(**GRIA1**-**GALNT10**-LARP1); **RGS6**; (**PDE4B-INSL5**); **NKAIN2**; **ANXA2**; **CTNNA3** HIPK3; FOXB1; ARNT2; ADAMTS14; DPP10; NREP; CACNA1C; SHISA6; SH3YL1; ACP1; TRDN; TSPAN18; INPP1
	Autism (17)	(**GRIA1**-**GALNT10**-LARP1); **RGS6**; **GRIN2B**; (**PDE4B-INSL5**); (ARL5B-**CACNB2**); **RERE; RBFOX1** IGSF9B; DCC; TBL1XR1; CACNA1C; CKB; IMPA2; ITIH3; ITIH4; GRM5

*Putative suicidality-related risk genes (PSRGs) are listed according to the phenotypes associated with them and the number of risk genes showing that phenotype is included in parentheses. Bold font indicates genes associated with several different clusters of phenotypes, whereas blue font indicates risk genes that were evaluated in the model of diminished motivation or immobility assay.

### PSRGs frequently interact with each other

Previously, network interactions among risk genes for other psychiatric disorders were found to be much more extensive than interactions among randomly-selected genes (33, 35). Consequently, we evaluated the interconnectivity of PSRGs versus randomly-selected genes with GeneMANIA. The PSRGs showed more extensive gene interactions with other members of the list compared with gene interactions among randomly-selected genes (**Supplementary Figure 1**). These findings support the contention that these genes are valid risk factors for STBs (36).

### Mutation of PSRGs in *C. elegans* produces relevant phenotype

**Figure 2:**
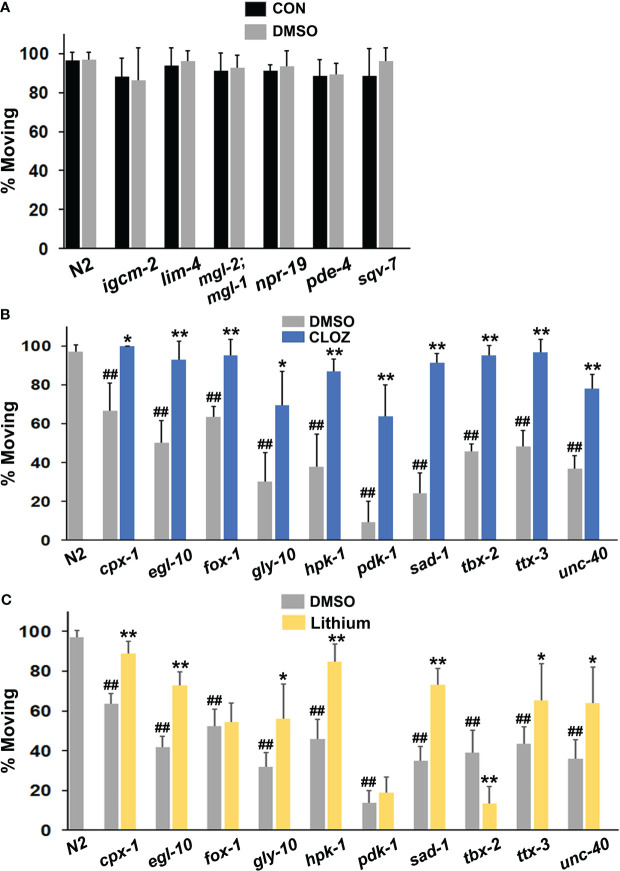
Evaluation of mutant animals in the immobility assay. **(A)** Wild-type N2 animals were assessed for movement (foraging) after removal from food on plates suffused with control (CON) buffer or with the same buffer containing a final concentration of 1% DMSO and were compared with various mutants (labelled on the x-axis). The *mgl-2;mgl-1* notation refers to a double mutant. Each bar in the figure represents at least 3 separate replicates of the experiment and the error bars depict the standard deviations **(A-C)**. Additional mutants were compared with N2 animals on plates suffused with DMSO or **(B)** clozapine (CLOZ) or **(C)** lithium at final concentrations of 160 μM or 6.7 mM, respectively. Significant differences from the N2 group are indicated by hash marks, ^##^p < 0.01. Ten of the 16 mutants tested showed immobility (diminished motivation) in the assay. For comparison, only 2 of 25 mutants displayed this phenotype in a separate study of insulin-related effects on recovery of pharyngeal pumping (58). A chi-square test revealed that the difference in the occurrence of the phenotype between the two studies was highly significant, p < 0.0002. For the mutants that showed immobility **(B, C)**, the level of movement on DMSO (baseline) was compared with movement on drug. Significant improvement of movement with a drug is indicated by asterisks: *p < 0.05, **p < 0.01.

We initially speculated that mutation of some PSRG counterparts in *C. elegans* would produce relevant phenotypes, including diminished motivation (immobility). Although the diminished motivation phenotype may be most relevant to depressive states, and not all depressed individuals consider suicide, it remained possible that certain suicide risk genes might be associated with disengagement from life activities. Six of the 17 strains evaluated were identical to the wild-type N2 strain with > 90% of animals moving 3 hours after transfer off bacteria (**Figure 2A**). Two of the mutants showing wild-type behavior did not have mutations in suicide risk genes per se, but had loss-of-function mutations in the *C. elegans* cannabinoid receptor (*npr-19*) and a transporter for UDP-N-acetylgalactosamine (*sqv-7*), which connects it to *gly-10*, an N-acetylgalactosaminyl transferase (see **Figure 2B**). It is worth noting that all of the mutant genes evaluated – regardless of whether they caused a phenotype – are expressed in the nervous system of *C. elegans*.

Remarkably, loss-of-function mutations in 10 of the 17 PSRGs produced significant immobility or diminished motivation to forage shortly after removal from food (**Figures 2B, C**). The frequency of this phenotype in strains with mutations in PSRGs significantly exceeds the frequency observed in a large number of mutants studied for their recovery of pharyngeal pumping following food deprivation (58). Generally, the absence or presence of DMSO did not affect the results (**Figure 2A**). Moreover, although two mutants (*unc-40* and *egl-10*) were sluggish, their immobility was not due to poor motor function because the immobility could be overcome by rapid touch of the tail or with drugs. Most animals continued to be immobile after 20 hr of food deprivation, despite still being sensitive to touch and drugs. Animals revert to normal active movement when returned to plates with food, so the effects are reversible. Overall, the failure of animals with mutations in PSRGs to forage in response to food deprivation appears to reflect motivational and not locomotor deficits.

Mutants from an additional strain, *glr-1(n2461)*, showed an interesting partial phenotype with normal rates of movement 3 hr after removal from food, but significant immobility after 20 hr of food deprivation that was corrected by a select panel of drugs (**Supplementary Figure 2**). GLR-1 is an ortholog of GRIA1/GRIA2 or alpha-amino-3-hydroxy-5-methyl-4 isoxazoleproprionic acid (AMPA) receptors.

### Drugs restore normal behavior in strains with mutations in PSRGs

Clozapine and lithium reduced suicidal behavior in controlled clinical studies (46–48). Therefore, we hypothesized that these drugs might decrease the immobility in some or all of the affected mutants if the corresponding human genes are relevant for STBs. The results in **Figure 2B** confirm that clozapine significantly reduced immobility in all of the mutants tested with full restoration of wild-type behavior in most of the mutants. Interestingly, lithium corrected immobility in most of the mutants, but not all of them (**Figure 2C**). In addition, lithium further decreased mobility (foraging) in *tbx-2* mutants, which indicates very different actions of this drug depending on the genetic makeup of the strain. This observation rules out the possibility of generalized, non-specific effects on motor activity.

**Figure 3:**
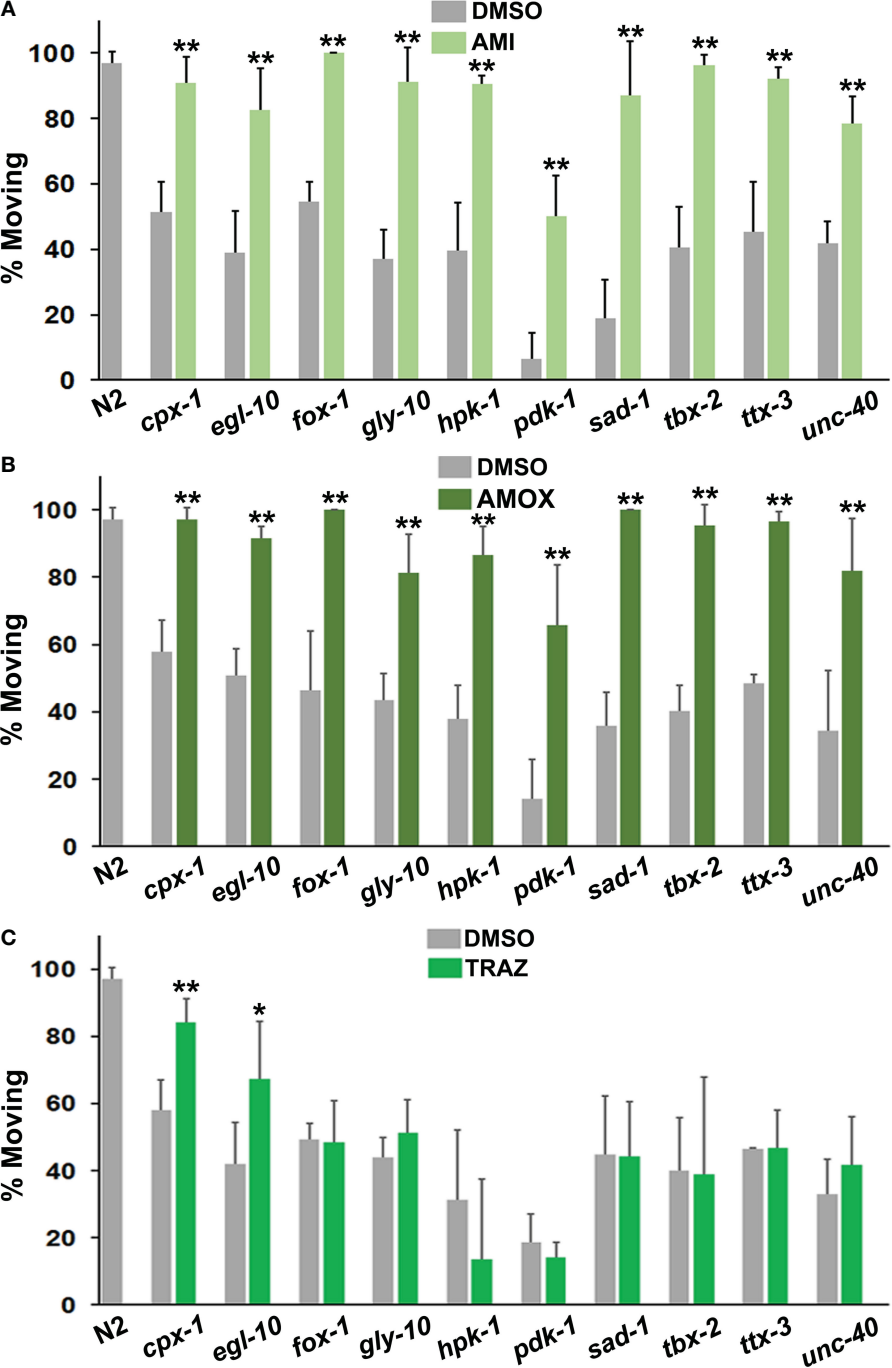
Comparison of responses to antidepressant drugs. The data are presented in the same way as in **Figure 2**. However, statistical comparisons between the mutants and the N2 group for DMSO conditions have not been indicated with hash marks. **(A)** Amitriptyline (AMI) was evaluated in the top panel, amoxapine (AMOX) in the middle **(B)** and trazodone (TRAZ) in panel **(C)**. These drugs were assayed at the same 160 μM concentration. All of the psychotropic drugs (antidepressants, antipsychotics, etc.) used in these studies have been tested with wild-type and other strains and none produce detectable changes in locomotion by themselves. Significant differences between DMSO and drug groups have been indicated with asterisks as before.

Older tricyclic antidepressants, amitriptyline and amoxapine, effectively corrected immobility in all of the mutants (**Figures 3A, B**) and they were very similar in terms of efficacy with the different strains. By contrast, trazodone only improved motivated behavior in two mutant strains (**Figure 3C**). Once again, the effectiveness of a drug was variable and depended upon the mutations present.

**Figure 4:**
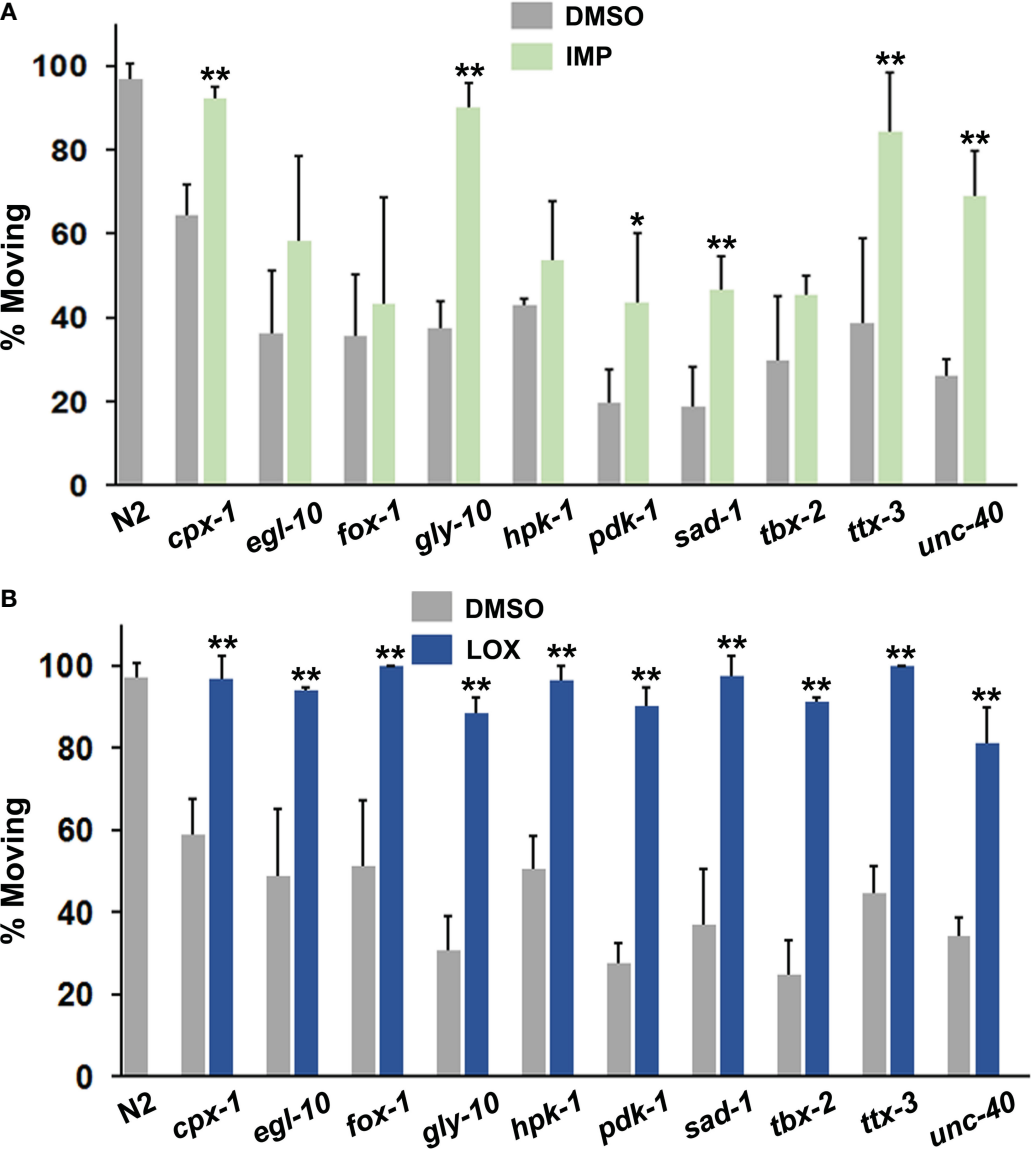
Evaluation of **(A)** imipramine (IMP) and **(B)** loxapine (LOX) for their ability to restore foraging (motivated) behavior. Both drugs were tested at 160 μM and significant differences have been indicated as before.

Imipramine behaved somewhat differently from amitriptyline and amoxapine. Overall, its effects were less pronounced in responsive mutants (**Figure 4A**). It also failed to correct immobility in *egl-10*, *fox-1*, *hpk-1* and *tbx-2* mutants. Previously, *egl-10* mutants have been reported to be resistant to the effects of imipramine on egg laying (59), which is consistent with the results presented here.

Based on structural and functional similarities to clozapine, we also tested loxapine for its effects in the immobility assay. As seen in **Figure 4B**, it was quite potent and significantly improved motivation to forage in all of the mutants evaluated, which was similar to clozapine. In addition to antagonizing dopamine D2 receptors, loxapine and clozapine inhibit serotonergic and muscarinic cholinergic receptors, which may be relevant to their effectiveness.

### Serotonergic and cholinergic systems mediate immobility induced by PSRGs

**Figure 5:**
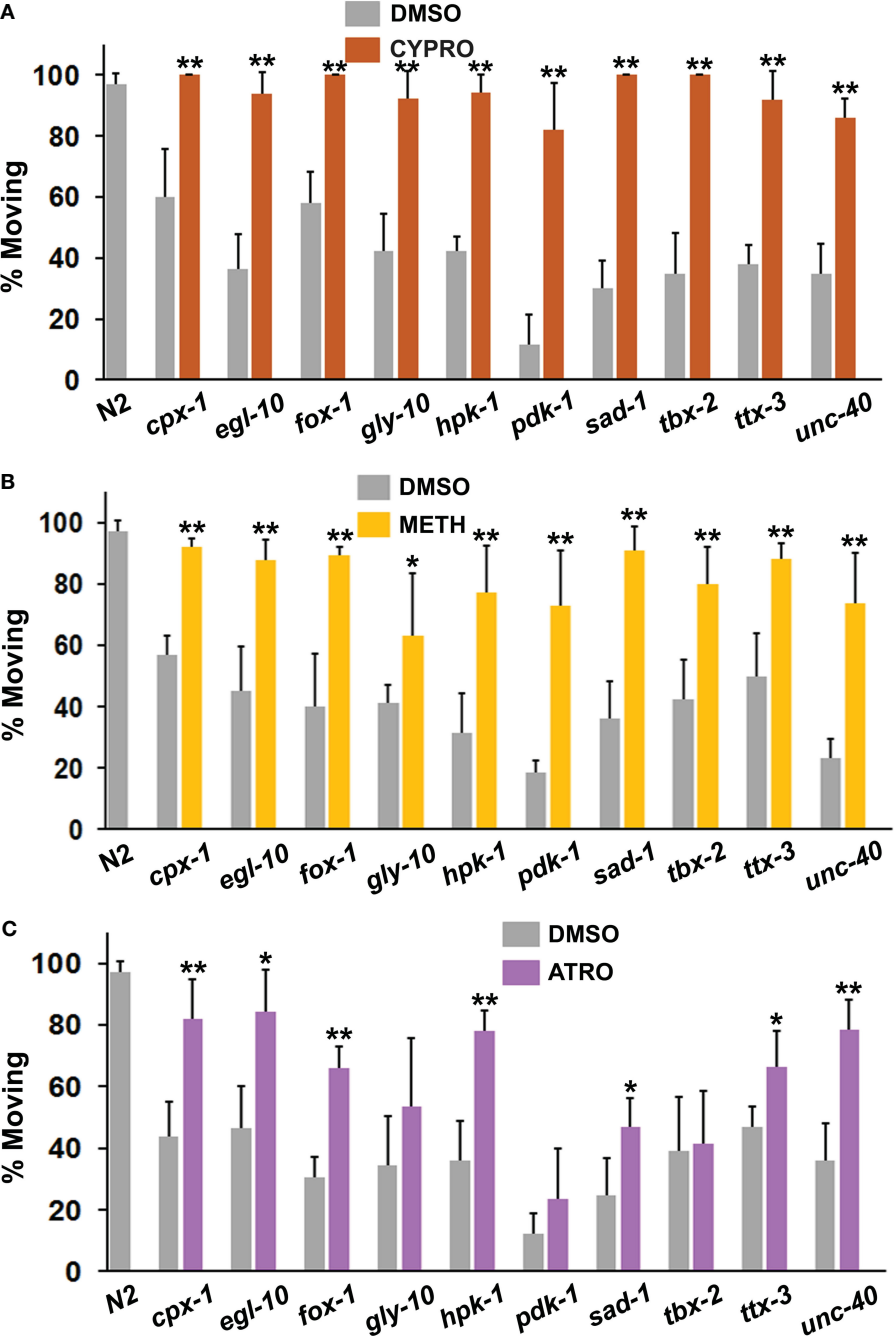
Assessment of serotonergic and muscarinic cholinergic antagonists for their ability to restore normal foraging behavior. Mutants were evaluated as described previously in the immobility assay in the presence of **(A)** cyproheptadine (CYPRO), **(B)** methiothepin (METH) or **(C)** atropine (ATRO). CYPRO and METH were tested at 160 μM, whereas atropine was used at a final concentration of 2 mM. Significant differences between DMSO and drug groups have been indicated with asterisks as before.

Although previous work implicated over-activity of serotonin and acetylcholine in the immobility response (40), the PSRGs may operate differently. To explore this possibility, we exposed animals to 5-HT2 receptor antagonists, cyproheptadine and methiothepin, and to atropine (muscarinic antagonist) prior to and during the immobility assay. Cyproheptadine was one of the most effective drugs at correcting the diminished motivation measured in the immobility assay (**Figure 5A**). All of the mutants showed a highly significant response to cyproheptadine and restoration of wild-type activity. A second 5-HT receptor antagonist, methiothepin, was similarly effective with all of the mutants (**Figure 5B**), which confirms an important role for serotonin in the motivational deficits. Atropine significantly reduced immobility in 7 of the strains, but not others (**Figure 5C**). Intriguingly, the main mutants (with the exception of *unc-40*) that responded well to atropine have been reported on WormBase to show altered sensitivity to aldicarb, an inhibitor of acetylcholinesterase.

## Discussion

In an effort to confirm and characterize possible suicide risk genes, we compiled a large and inclusive list of implicated genes and determined that they are evolutionarily conserved, enriched for genes that are essential for life and are highly networked with each other. Additionally, we identified common themes among the phenotypes associated with the PSRGs including psychiatric symptoms, metabolic outcomes and asthma/allergy. Moreover, these studies revealed that mutation of the PSRGs caused a relevant phenotype in *C. elegans* that was corrected by drugs shown clinically to reduce suicidal behavior. These findings are significant because they increase our confidence that these genes should qualify as bona fide suicide risk genes. Furthermore, they implicate certain signaling pathways (serotonin and insulin) and fundamental behaviors such as diminished goal-directed motivation in the emergence of STBs.

The immobility assay is a measure of drive or motivation to search for food in response to food deprivation. The absence of goal-directed behavior in animals fully capable of locomotion has been interpreted to reflect diminished motivation (40). Diminished motivation to engage in life activities (known as anhedonia) is a hallmark feature of major depression and other psychiatric disorders and is also akin to the hopelessness or despair underlying suicidal ideation and behavior (60). Elsewhere, we have discussed in detail how diminished motivation contributes to STBs (42).

Foraging or area restricted search has been characterized previously and is mediated neurochemically by serotonin (61), dopamine/glutamate (62), acetylcholine and insulin (40). Cyproheptadine and methiothepin effectively corrected immobility in all of the mutants tested, whereas atropine was effective in 7 of 10 mutant strains. Curiously, drugs that inhibit serotonin reuptake, such as amitriptyline, imipramine and trazodone, also improved motivated behavior in some of the mutants. Of course, the pharmacology of these drugs is complex because amitriptyline and imipramine also antagonize muscarinic receptors, whereas trazodone inhibits 5-HT2 and α-adrenergic receptors. The pharmacology of the response in animals with mutations in PSRGs is consistent with previous studies (40) and implicates an excess of serotonin and acetylcholine in diminished motivation. The role of serotonin in depression has always been complicated. Early studies found that individuals with depression tended to show either very low or high levels of the serotonin metabolite 5-hydroxyindoleacetic acid (5-HIAA) in CSF that also predicted suicide attempts (63). Furthermore, 5-HT2 receptor antagonists also treat depressive symptoms (64, 65). This complicated biology of serotonin is consistent when we recognize its major role as a mediator of homeostasis (66).

Another striking feature of the data is the apparent interaction between drug effects and genetic mutations. Consequently, certain drugs did not correct immobility in all of the mutants despite the strains being essentially identical genetically outside of the primary mutant allele. In particular, lithium, atropine, imipramine and trazodone were effective at improving motivation in only some of the mutants tested. The diversity of responses by the mutants to the panel of drugs and the comparable effects of different serotonergic antagonists strongly argue against non-specific effects in our system. The variability in responsiveness could occur because some of the mutations affect pathways that the drug can effectively address, whereas other mutations affect different pathways that the drug cannot correct. Alternatively, a mutant may not respond to a drug because the mutation directly affects the receptor, pathway, etc. targeted by that drug. For example, the failure of lithium to correct immobility in the *pdk-1* mutants may be due to the fact that lithium can act via a signaling pathway that requires the insulin receptor and *pdk-1* (67). This line of research might yield insights into genetic factors controlling therapeutic responses to drugs.

The broad effects of psychotropic drugs may complicate interpretation of the findings, although this may ultimately allow greater understanding of their mechanism of action. At first glance, it may seem surprising that some of the drugs rescued the phenotype in most or all of the mutants tested. However, we typically evaluated two or more from a single class, for example clozapine and loxapine (antipsychotics) and amitriptyline, amoxapine and imipramine (antidepressants), there was some pharmacological overlap (e.g., anticholinergic activity), and the drugs are structurally quite similar (**Supplementary Figure 3**). Selective actions of the drugs are indicated by the fact that no discernible effects on locomotion were observed for clozapine, loxapine and amitriptyline in wild-type animals (40), and clozapine and lithium did not affect the movement phenotype of another mutant – *unc-77(e625)* (68). Moreover, the simplicity of *C. elegans* means there may be a limited number of mechanisms – possibly interconnected – that mediate immobility across the various mutants in response to food deprivation. Clozapine and loxapine – two of the most effective drugs – inhibit an array of monoaminergic and cholinergic receptors in addition to promoting Akt activation (69, 70). Older tricyclic antidepressants such as amitriptyline and amoxapine also block muscarinic cholinergic receptors, which is similar to atropine, an active drug in our assay. The anticholinergic drug scopolamine shows rapid antidepressant effects (71). Similarly, when imipramine was first introduced, it treated anhedonia and suicidal tendencies within a few days (72). At that time, it was used at higher doses that routinely produced anticholinergic effects such as dry mouth, etc. (72), which might explain the quicker onset of therapeutic benefit.

Taken together, analysis of the phenotypes and pathways implicated by the mutants suggests relevant functional connections to STBs. Multiple PSRGs were associated with diabetes (17) and asthma (15). Diabetes has been linked to increased apathy [diminished motivation (43)], depression (44, 73) and suicidal ideation and attempts (45, 74). Likewise, there is a 2.5-4-fold increased risk of suicide attempts in patients with asthma (75, 76). The present studies confirm that motivation to forage is regulated by insulin, serotonin and acetylcholine. Furthermore, these three signaling molecules are linked to asthma (77, 78), so the overlap is likely to be meaningful.

Several mutants clearly fit this story because they affect insulin signaling and dauer or lifespan in *C. elegans*, including *pdk-1*, *tbx-2*, *sad-1*, and *hpk-1*. Other PSRGs have been implicated in regulating synaptic responses and neuronal excitability including *egl-10* (RGS6), *cpx-1* (CPLX1), and *fox-1* (RBFOX1) (79–82), which is consistent with the findings of Sokolowski et al. (25). Loss of additional synaptic components, namely AMPA receptors, mediates the delayed immobility phenotype displayed by *glr-1* mutants, which might serve as a useful model for chronic stress/exhaustion (83). Finally, Mealer et al. (84) linked GALNT10 (*gly-10*) to defects in glycosylation in schizophrenia – it may be needed to glycosylate key neuronal proteins. Thus, a picture begins to emerge of how the PSRGs act at the molecular level to potentially affect neuronal mediation of motivated behavior.

Additional findings revealed that PSRGs participated in more gene-gene interactions than randomly-selected genes. They may be more interactive because they are evolutionarily-conserved, which means a longer residence time in the genome, with the potential for more extensive integration into networks (35). This observation has previously been cited as providing additional validation for the authenticity of risk genes for bipolar disorder (36).

Limitations of this study include the lack of independent confirmation of many of the genetic variants as bona fide risk factors for suicide. Of course, the studies reported here were aimed at addressing this issue by providing functional validation of a selection of risk genes. Moreover, the list of PSRGs is not comprehensive, in part, because new risk genes are still being identified, which tempers interpretation of the findings. The PSRG data were derived from a spectrum of subjects with suicidal behavior and/or suicidal ideation across several diagnoses. A more restricted focus, e.g., on subjects with suicide attempts and depression, may alter the findings to some degree; however, we sought to analyze the broadest set of data because suicide is a transdiagnostic phenomenon. Some of the differences in responsiveness to drugs between mutants could have emerged because not all of the strains have been backcrossed allowing for contributions by unknown mutations in other genes. However, this seems unlikely because consistent patterns emerged when comparing mutant strains and their responses to drugs and the low probability that random mutations will affect motivation in multiple mutants. Furthermore, diminished motivation, as measured in the immobility assay, could be interpreted in different ways than our view. For example, the immobility could also reflect a depressive-like state that is tilted toward suicidal actions by disinhibiting factors such as alcohol use. Nevertheless, it is striking that mutations in so many of the PSRGs caused a potentially relevant phenotype at a rate that appears to defy coincidence.

Since the initial completion of this work, a meta-analysis of GWAS on suicide risk genes has recently been published by Docherty et al. (85). The analysis strongly endorsed one of the genes evaluated here, PDE4B, and 6 others: DRD2, SLC6A9, FURIN, NLGN1, SOX5 and CACNG2. Five of these PSRGs have notable connections to the molecular mechanisms implicated by our findings. DRD2 is the target of antipsychotic drugs that rescue immobility and that activate Akt in the insulin-signaling pathway (70). The *C. elegans* counterpart of FURIN, *kpc-1*, cleaves insulin precursors to produce active proteins (86), whereas *egl-13*, the ortholog of SOX5, regulates neuronal cell fate, including development of a major insulin-secreting neuron, AIA (87). The CACNG2 gene product regulates AMPA receptors such as *glr-1* (88) that produced a delayed immobility phenotype when mutated, while the NLGN1 ortholog, *nlg-1*, functionally interacts with *unc-40* (89), which has been characterized here. Overall, the new data lend additional support for the genes and pathways identified in our study.

Future studies will need to evaluate additional PSRGs for their effects on motivation as well as other suitable phenotypes both in *C. elegans* and other model systems. The possibility that diminished motivation as measured in the immobility assay constitutes a protophenotype (39) for aspects of suicidal behavior in man merits serious consideration. Finally, the PSRGs characterized here should be considered meaningful leads for learning more about the genetic and mechanistic contributions to suicide.

## Data availability statement

The original contributions presented in the study are included in the article/**Supplementary Material**. Further inquiries can be directed to the corresponding author.

## Ethics statement

The manuscript presents research on animals that do not require ethical approval for their study.

## Author contributions

TA: Investigation, Methodology, Writing – review & editing. AT: Investigation, Methodology, Writing – review & editing. MN: Data curation, Investigation, Methodology, Software, Writing – review & editing. LH: Investigation, Methodology, Writing – review & editing. AZ: Investigation, Methodology, Writing – review & editing. DD: Conceptualization, Investigation, Methodology, Project administration, Supervision, Writing – original draft.
