# Supplementary material for: Evolutionary conservation of putative suicidality-related risk genes that produce diminished motivation corrected by clozapine, lithium and antidepressants
Source: Front Psychiatry. 2024 Feb 1;15:1341735. doi: 10.3389/fpsyt.2024.1341735 (PMC10867104; doi:10.3389/fpsyt.2024.1341735)

**Supplementary Materials**

**Evolutionary conservation of putative suicidality-related risk genes that produce diminished motivation corrected by clozapine, lithium and antidepressants**

Titilade Ajayi, Alicia Thomas, Marko Nikolic, Lauryn Henderson, Alexa Zaheri and Donard S. Dwyer

**Content**

Supplementary Methods

Supplementary References

Supplementary Figure Legends

Supplementary Fig. 1. Genetic interactions among risk genes.

Supplementary Fig. 2. Delayed onset immobility in *glr-1* mutants.

Supplementary Fig. 3. Structural similarities between rescue drugs.

**SUPPLEMENTARY METHODS**

**Immobility assay and drug studies**

At the initiation of this study, we sought to determine if any of the mutant strains showed immobility in the foraging assay when exposed to dilute acetic acid (baseline condition) and dimethyl sulfoxide (DMSO) – a required component in the original experiments Dagenhardt et al., 2017). Animals were grown on large nematode growth medium (NGM) plates with 5X peptone plus bacteria and the plates were then transferred from 20 °C to 25 °C in an incubator overnight. This step was required for temperature-sensitive strains in the original studies and was kept as a condition because the elevated temperature biases animals toward movement, which then requires robust gene effects to produce immobility. Consequently, animals were kept at 25 °C for all stages of the experiment. Young adult animals were then transferred individually to small culture plates (20-25 per plate) containing bacteria and dilute acetic acid, dilute acetic acid with DMSO (1% final concentration on the plates) or drug diluted in DMSO (in subsequent studies). They were incubated for 90 min before removal from food. For the immobility assay, the animals were next transferred to plates with no food that included buffer or buffer plus DMSO in the absence or presence of drug as in the prior step. Animals were examined for movement 2.5-3 hr later and in some cases after 20 hr. Strains that showed the immobility phenotype in this assay were then tested to determine if drugs would correct the diminished motivation.

For drug rescue studies, pharmacological agents were purchased from Millipore-Sigma-Aldrich or Bio-Techne Corp. The choice of drug concentration was based on several factors. Most of the drugs have been studied previously in *C. elegans* and produced physiological effects at the same concentrations used here (Dagenhardt et al., 2017; Donohoe et al., 2006; Karmacharya et al., 2009; McCool et al., 2008; Weeks et al., 2010). Concentrations for other drugs (e.g., trazodone) were in the same range or less than that used previously for similar dugs, e.g., fluoxetine (Kullyev et al., 2010). As reported by other groups, drug concentrations in the worm are generally about one-hundredth of that applied to the plate (Matta et al., 2007; McColl et al., 2008; Weinshenker et al., 1995). The affinity of these drugs for *C. elegans* monoamine and serotonergic receptors is in the range of 25-100 nM when drugs bind directly to receptors expressed in cells (Hobson et al., 2003; Smith et al., 2007); however, it requires 50-200 μM to observe behavioral changes in animals via these same receptors when drug exposure occurs via agar plates (Dagenhardt et al., 2017; Donohoe et al., 2006; Karmacharya et al., 2009; Kullyev et al., 2010; McColl et al., 2008; Weeks et al., 2010; Weinshenker et al., 1995). This disparity is attributed to the cuticle barrier, uptake/absorption issues and distribution effects.

After 90 min on the initial control/drug plates, animals were transferred individually to large, bacteria-free NGM plates (no peptone) previously suffused with DMSO (control) or drug at the desired concentration. Normally in the absence of bacteria, animals increase activity and continuously search for food. With certain genetic mutations, the animals cease moving after about 10-30 min and only make short, periodic movements over the next several days (Dagenhardt et al., 2017). After 2.5-3 hr on the plates without bacteria, we checked for immobility. Animals were observed individually for 5 sec to detect movement and were considered to be moving if they traversed forward or backward more than half their body length. We counted the number moving out of the total number of animals on the plate to derive the % Moving. We were careful not to jostle the plates and used relatively low illumination to observe movement to avoid disturbing animals in their baseline condition. To guard against effects of occasional injury during transfer, animals were only counted as immobile if they responded to touch on the tail (after the 5-sec observation period) with a rapid escape response.

**SUPPLEMENTARY REFERENCES**

Dagenhardt, J., Trinh, A., Sumner, H., Scott, J., Aamodt, E., Dwyer, D.S., 2017. Insulin signaling deficiency produces immobility in *C. elegans* that models diminished motivation states in man and responds to antidepressants. Mol. Neuropsychiatry 3, 97–107. doi:10.1159/000478049

Donohoe, D.R., Aamodt, E.J., Osborn, E., Dwyer, D.S., 2006. Antipsychotic drugs disrupt normal development in *Caenorhabditis elegans* via additional mechanisms besides dopamine and serotonin receptors. Pharmacol. Res. 54, 361-372.

Hobson, R.J., Geng, J., Gray, A.D., Komuniecki, R.W., 2003. SER-7b, a constitutively active Gα_s_ coupled 5-HT_7_-like receptor expressed in the *Caenorhabditis elegans* M4 pharyngeal motorneuron. J. Neurochem. 87, 22-29. doi:10.1046/j.1471-4159.2003.01967.x

Karmacharya, R., Sliwoski, G., Lundy, M., Suckow, R.F., Cohen, B.M., Buttner, E.A., 2009. Clozapine interaction with phosphatidyl inositol 3-kinase (PI3K)/insulin-signaling pathway in *Caenorhabditis elegans*. Neuropsychopharmacology 34, 1968–1978. doi:10.1038/npp.2009.35

Kullyev, A., Dempsey, C.M., Miller, S., Kuan, C.J., Hapiak, V.M., Komuniecki, R.W., Griffin, C.T., Sze, J.Y., 2010. A genetic survey of fluoxetine action on synaptic transmission in *Caenorhabditis elegans*. Genetics 186, 929-941. doi:10.1534/genetics.110.118877

Matta, S.G., Balfour, D.J., Benowitz, N.L., Boyd, T., Buccafusco, J.J., Caggiula, A.R., Craig, C.R., Collins, A.C., Damaj, M.I., Donny, E.C., Gardiner, P.S., Grady, S.R., Heberlein, U., Leonard, S.S., Levin, E.D., Lukas, R.J., Markou, A., Marks, M.J., McCallum, S.E., Parameswaran, N., Perkins, K.A., Picciotto, M.R., Quik, M., Rose, J.E., Rothenfluh, A., Schafer, W.R., Stolerman, I.P., Tyndale, R.F., Wehner, J.M., Zirger, J.M., 2007. Guidelines on nicotine dose selection for in vivo research. Psychopharmacology 190, 269-319. doi:10.1007/s00213-006-0441-0

McColl, G., Killilea, D.W., Hubbard, A.E., Vantipalli, M.C., Melov, S., Lithgow, G.J., 2008. Pharmacogenetic analysis of lithium-induced delayed aging in *Caenorhabditis elegans*. J. Biol. Chem. 283, 350–357. [doi:10.1074/jbc.M705028200](https://doi.org/10.1074/jbc.M705028200)

Smith, K.A., Rex, E.B., Komuniecki, R.W., 2007. Are *Caenorhabditis elegans* receptors useful targets for drug discovery: pharmacological comparison of tyramine receptors with high identity from *C. elegans* (TYRA-2) and *Brugia malayi* (Bm4). Mol. Biochem. Parasitol. 154, 52-61. doi:10.1016/j.molbiopara.2007.04.004

Weeks, K.R., Dwyer, D.S., Aamodt, E.J., 2010. Antipsychotic drugs activate the *C. elegans* Akt pathway via the DAF-2 Insulin/IGF-1 Receptor. ACS Chem. Neurosci. 1, 463-473. doi:10.1021/cn100010p

Weinshenker, D., Garriga, G., Thomas, J.H., 1995. Genetic and pharmacological analysis of neurotransmitters controlling egg laying in *C. elegans*. J. Neurosci. 15, 6975-6985.

**SUPPLEMENTARY FIGURE LEGENDS AND FIGURES**

**Supplementary Fig. 1. Genetic interactions among risk genes.** The results of GeneMANIA analysis of 105 randomly-selected genes and the interaction network obtained with the PSRGs (right panel). Not all of the genes from the two lists were involved in interactions as compiled in the GeneMANIA database and have been omitted for clarity. The density of interactions is depicted here and the number of links per gene was calculated automatically and is shown in the lower panel. Gene interaction data were obtained with 4 separate lists of 105 randomly-selected genes, the average number of links per gene was calculated and the standard deviation (SD) of these data was determined to derive the confidence interval shown by the gold line. The line represents 4 X the SD, which was 0.39 indicating low variability for the control data. Asterisks (**) indicate significant differences between the two groups, p < 0.0001.

**Supplementary Fig. 1**


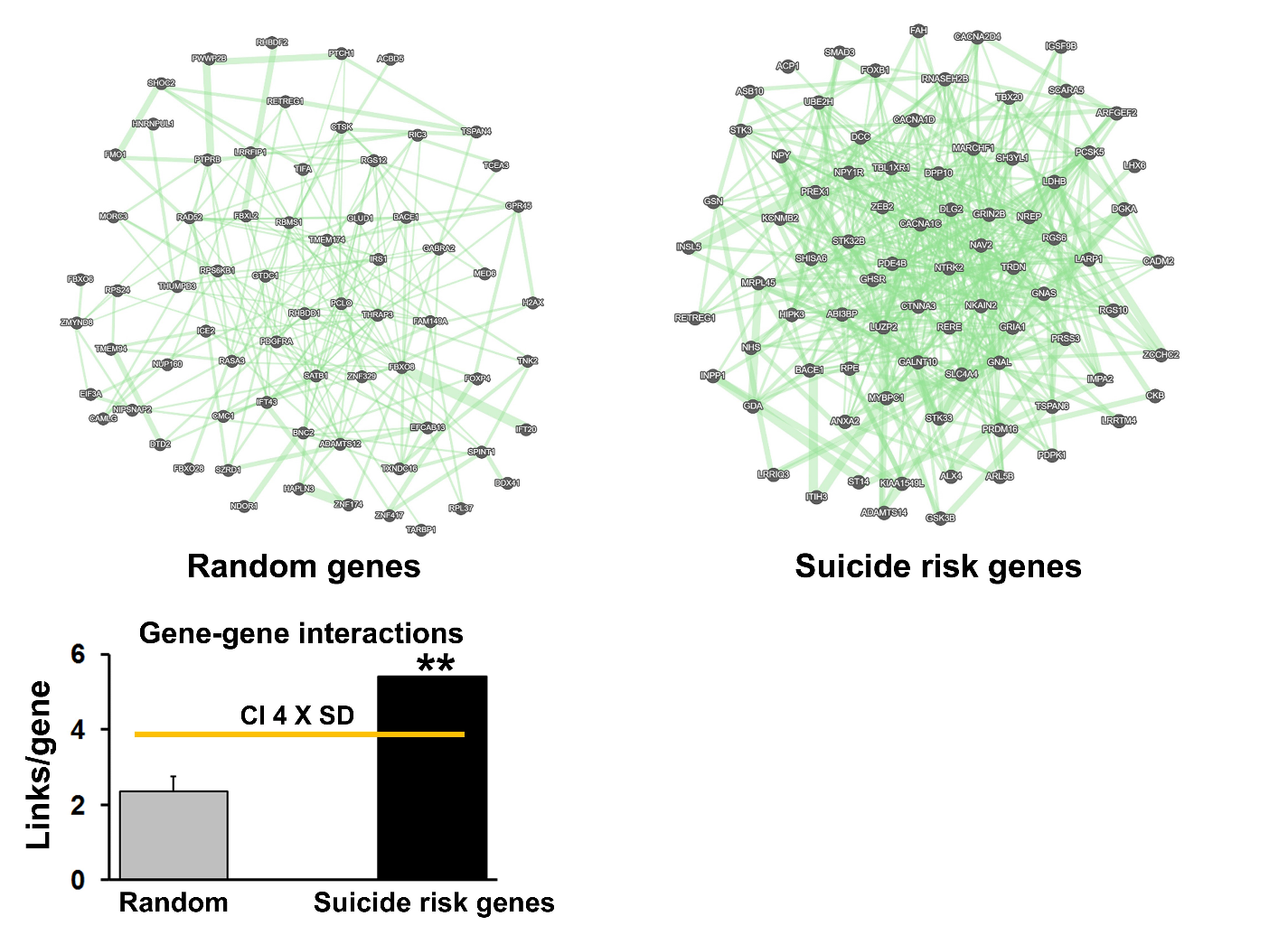


**Supplementary Fig. 2. Delayed onset immobility in *glr-1* mutants**. (**a**) Wild-type (N2) and *glr-1* animals were evaluated for movement in the immobility assay after 2 h and 20 h of food deprivation (labeled). For the assay, plates contained either dilute acetic acid as a control (CON; black bars) or a final concentration of 1% DMSO (gray bars). There was a significant reduction in goal-directed behavior in *glr-1* mutants at 20 h under both buffer conditions. The immobile *glr-1* animals still responded to touch with a vigorous escape response at the 20 h-time point. Therefore, physical exhaustion does not explain the failure to search for food or disperse. Each experiment was repeated at least 3 times with 20-25 animals added per plate and the asterisks indicate *p < 0.05 and **p < 0.01. (**b**) Various drugs (labeled) were tested for their ability to restore goal-directed behavior at the 20 h-time point. Clozapine (CLOZ), amitriptyline (AMI), amoxapine (AMOX), cyproheptadine (CYPRO) and imipramine (IMP) were used at a concentration of 160 μM, whereas lithium was used at 6.7 mM as before. Each experiment was repeated at least 3 times and means and standard deviations are shown here. Significant improvement of movement compared with the DMSO only condition is indicated by asterisks - **p < 0.01. Lithium and imipramine failed to increase movement at 20 h.

**Supplementary Fig. 2**


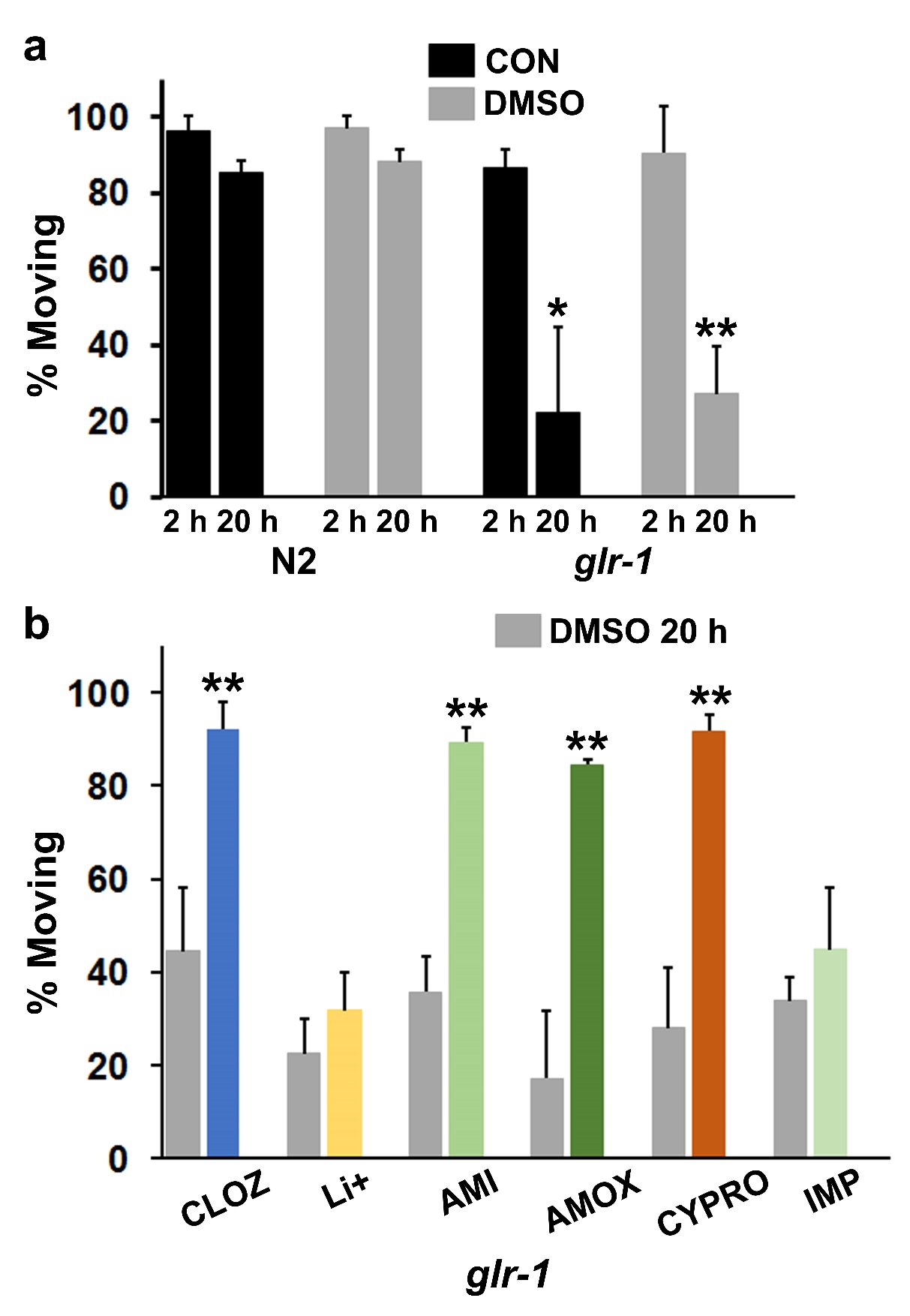


**Supplementary Fig. 3. Structural similarities between rescue drugs.** Below are the chemical structures of the drugs that produced the greatest rescue of the immobility phenotype. Similarities include tricyclic ring structures and attached piperazine moieties. Clozapine and loxapine differ mainly in the nitrogen vs. oxygen atom in the central ring, whereas loxapine and amoxapine differ by only a single methyl group.

**Supplementary Fig. 3**

**Clozapine Loxapine Amoxapine**


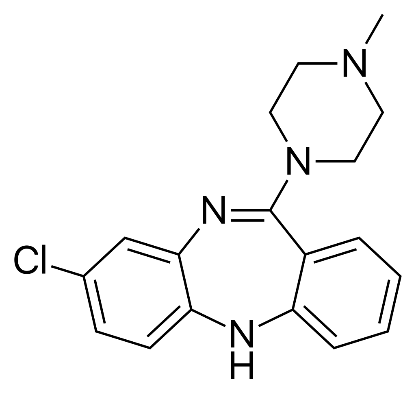

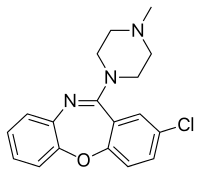

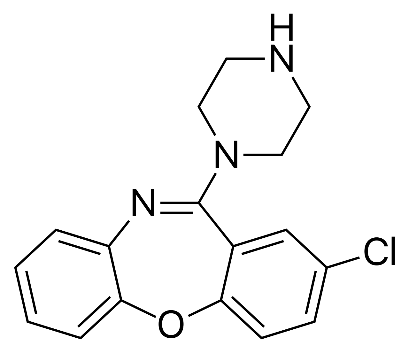


**Amitriptyline Cyproheptadine Methiothepin**


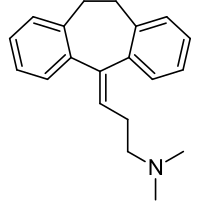

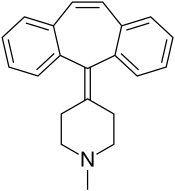

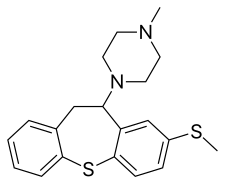

Supplement: Supplementary file 2 [file DataSheet_1.docx]
